# Use of the Adaptive LASSO Method to Identify PM_2.5_ Components Associated with Blood Pressure in Elderly Men: The Veterans Affairs Normative Aging Study

**DOI:** 10.1289/ehp.1409021

**Published:** 2015-06-19

**Authors:** Lingzhen Dai, Petros Koutrakis, Brent A. Coull, David Sparrow, Pantel S. Vokonas, Joel D. Schwartz

**Affiliations:** 1 Department of Environmental Health, and; 2 Department of Biostatistics, Harvard T.H. Chan School of Public Health, Boston, Massachusetts, USA; 3 Veterans Affairs Normative Aging Study, Veterans Affairs Boston Healthcare System, Department of Medicine, Boston University School of Medicine, Boston, Massachusetts, USA

## Abstract

**Background:**

PM_2.5_ (particulate matter ≤ 2.5 μm) has been associated with adverse cardiovascular outcomes, but it is unclear whether specific PM_2.5_ components, particularly metals, may be responsible for cardiovascular effects.

**Objectives:**

We aimed to determine which PM_2.5_ components are associated with blood pressure in a longitudinal cohort.

**Methods:**

We fit linear mixed-effects models with the adaptive LASSO penalty to longitudinal data from 718 elderly men in the Veterans Affairs Normative Aging Study, 1999–2010. We controlled for PM_2.5_ mass, age, body mass index, use of antihypertensive medication (ACE inhibitors, non-ophthalmic beta blockers, calcium channel blockers, diuretics, and angiotensin receptor antagonists), smoking status, alcohol intake, years of education, temperature, and season as fixed effects in the models, and additionally applied the adaptive LASSO method to select PM_2.5_ components associated with blood pressure. Final models were identified by the Bayesian Information Criterion (BIC).

**Results:**

For systolic blood pressure (SBP), nickel (Ni) and sodium (Na) were selected by the adaptive LASSO, whereas only Ni was selected for diastolic blood pressure (DBP). An interquartile range increase (2.5 ng/m^3^) in 7-day moving-average Ni was associated with 2.48-mmHg (95% CI: 1.45, 3.50 mmHg) increase in SBP and 2.22-mmHg (95% CI: 1.69, 2.75 mmHg) increase in DBP, respectively. Associations were comparable when the analysis was restricted to study visits with PM_2.5_ below the 75th percentile of the distribution (12 μg/m^3^).

**Conclusions:**

Our study suggested that exposure to ambient Ni was associated with increased blood pressure independent of PM_2.5_ mass in our study population of elderly men. Further research is needed to confirm our findings, assess generalizability to other populations, and identify potential mechanisms for Ni effects.

**Citation:**

Dai L, Koutrakis P, Coull BA, Sparrow D, Vokonas PS, Schwartz JD. 2016. Use of the adaptive LASSO method to identify PM_2.5_ components associated with blood pressure in elderly men: the Veterans Affairs Normative Aging Study. Environ Health Perspect 124:120–125; http://dx.doi.org/10.1289/ehp.1409021

## Introduction

Studies have shown that exposure to fine particulate matter (PM_2.5_; particles ≤ 2.5 μm in aerodynamic diameter) is associated with cardiovascular morbidity and mortality (Franklin et al. 2008; Laden et al. 2006; Miller et al. 2007; Zanobetti et al. 2009). PM_2.5_ consists of various components, including organic and elemental carbon, metals, and ions. Some national studies have evaluated whether PM_2.5_ components may have differential effects on cardiovascular health (Dai et al. 2014; Peng et al. 2009), but it still is not clear whether specific components may be responsible for PM_2.5_-related cardiovascular effects.

Increased blood pressure is a major risk factor for cardiovascular events. Several studies have investigated the relationship between PM and blood pressure. However, the results have varied, possibly because of differences in the particle composition (Baccarelli et al. 2011; Choi et al. 2007; Chuang et al. 2010; Dvonch et al. 2009; Harrabi et al. 2006; Hoffmann et al. 2012; Ibald-Mulli et al. 2004; McCracken et al. 2007; Schwartz et al. 2012; Wilker et al. 2009, 2010).

Inhaled PM-associated metals may be able to translocate from lung into systemic circulation and induce adverse effects on cardiovascular system (Wallenborn et al. 2007). There is growing evidence supporting adverse effects of ambient metals on cardiovascular health. For example, iron (Fe), potassium (K), titanium (Ti), and zinc (Zn) in fine particles were positively associated with cardiovascular mortality in a California study (Ostro et al. 2007). A multiple-community study reported that Ni and Na^+^ modified associations of PM_2.5_ on hospital admissions due to cardiovascular diseases (Zanobetti et al. 2009). Also, numerous animal studies have reported cardiovascular toxicity of PM metal components Zn, Ni, and vanadium (V) (Campen et al. 2001; Chuang et al. 2013; Kodavanti et al. 2008; Lippmann et al. 2006). In terms of sources, PM-associated metals usually come from road dust [e.g., calcium (Ca), aluminum (Al), Fe, Ti], oil combustion (e.g., Ni, V), traffic emission [e.g., Zn, copper (Cu)], wood burning (e.g., K), and sea salt (e.g., Na).

In this study, we examined the association between blood pressure and 11 PM_2.5_ components, including 8 metals (Fe, K, Al, Ni, V, Cu, Zn, and Na) and 3 nonmetals [sulfur (S), silicon (Si), and selenium (Se)], with longitudinal data from the Veterans Affairs Normative Aging Study.

## Methods

*Study population.* The Normative Aging Study (NAS) was established in 1963 by the Department of Veterans Affairs (Bell et al. 1972). Briefly, it is an ongoing longitudinal study of aging, which enrolled 2,280 community-dwelling, healthy men living in the Greater Boston, Massachusetts, area. Participants were free of known chronic medical conditions at enrollment and have undergone examinations every 3 to 5 years, including physical examinations and questionnaires. All participants provided written informed consent. The study was reviewed and approved by the institutional review boards of all participating institutions.

After we excluded participants with incomplete information for any of the covariates of interest, those who died, or those who moved out of New England, a total of 718 participants with 1,567 observations had examinations between March 1999 and October 2010. Of the 718 participants, 235 (33%) had one visit, 195 (27%) had two visits, and 288 (40%) had three or more visits.

*Blood pressure measurements.* During a clinical visit, a physician uses a standard mercury sphygmomanometer with a 14-cm cuff to measure blood pressure for the subject while he is sitting, including systolic blood pressure (SBP) and fifth-phase diastolic blood pressure (DBP) in each arm to the nearest 2 mmHg. We used the means of the left and right arm measurements as a subject’s SBP and DBP.

*Environmental data.* Daily ambient PM_2.5_ and its components were measured at the stationary ambient monitoring site at the Harvard University Countway Library (Kang et al. 2010), using the tapered element oscillating microbalance (TEOM 1400a; Rupprecht & Patashnick Co.) and the energy dispersive X-ray fluorescence spectrometer (Epsilon 5; PANalytical), respectively. The monitoring site is 1 km from the clinical examination site.

We obtained daily temperature data from Boston Logan airport weather station.

*Statistical analysis.* We used 7-day moving-average concentrations for PM_2.5_ and the 11 components—K, S, Se, Al, Si, Fe, Ni, V, Cu, Zn, and Na—because previous studies have suggested that PM averaging over that time period is strongly associated with blood pressure (Mordukhovich et al. 2009; Wilker et al. 2010; Zanobetti et al. 2004). We focused on these components because their concentration levels are mostly above the method detection limits and they are representative of different PM sources (Hopke et al. 2006). In the analysis, we controlled for continuous variables age, body mass index [BMI; computed as weight (in kilograms) divided by height (in square meters)], years of education, linear and quadratic terms of mean temperature of visit day, and categorical variables use of each individual type of antihypertensive medication (ACE inhibitors, non-ophthalmic beta blockers, calcium channel blockers, diuretics, and angiotensin receptor antagonists), smoking status (three categories: never, former, current smoker), alcohol intake (whether the participant takes two or more drinks per day; yes or no), and season (four categories; defined as spring: March–May, summer: June–August, fall: September–November, winter: December–February) regardless of statistical significance because these variables have been shown to predict cardiovascular health (Mordukhovich et al. 2009; Schwartz et al. 2012). In addition, we adjusted for potential confounding of associations with PM_2.5_ components by PM_2.5_ mass (Mostofsky et al. 2012). All variables were measured at each visit. We forced these covariates to be included in the models and estimated their fixed effects with no penalization.

Selecting important predictors from a large list of correlated predictors is difficult, and most methods are empirical. Approaches such as stepwise methods ignore stochastic errors inherited in the stages of variable selection (Fan and Li 2001) and can yield falsely narrow confidence intervals (Harrell 2001). To improve on this, we applied the adaptive LASSO (least absolute shrinkage and selection operator) method to select important component(s) that may be associated with blood pressure from those 11 PM_2.5_ components. Briefly, the LASSO is a regression shrinkage and selection approach that applies an *l*_1_ penalty to the component regression coefficients. This penalty essentially minimizes the sum of squared errors subject to the sum of the absolute values of the coefficients being less than a given value (Tibshirani 1996). The adaptive LASSO is a later version of the LASSO, which uses weights for penalizing different coefficients in the *l*_1_ penalty and enjoys the oracle properties, which means, given that the true model depends only on a subset of the predictors, this selection procedure is able to identify the right subset model and satisfies asymptotic normality (Fan and Li 2001; Zou 2006). Because subjects had repeated measures, we fit linear mixed-effects models with random subject-specific intercepts to capture the correlation among different measurements within the same subject, as follows:

*Y_i_ =* ***X****_i_***α** *+* ***Z****_i_***β** *+ μ_i_ +* ε*_i_*, [1]

where, *Y_i_* is the blood pressure level (SBP or DBP) of subject *i*, ***X****_i_ = (X_i1_, …, X_iP_)^T^* is a vector of PM_2.5_ mass and other covariates, ***Z****_i_ = (Z_1i_,…,Z_iM_)^T^* is a vector of PM_2.5_ components, *μ_i_* is the random intercept. Hence, **α** indicates the fixed effects of PM_2.5_ mass and other covariates ***X****_i_*, and **β** is the penalized effects of PM_2.5_ components ***Z****_i_* that are given by the adaptive LASSO.

First, we used the ordinary linear mixed-effects (LME) model to obtain non-zero coefficients (β*_lme_*) for each component, and computed the adaptive weight as its inverse (*w* = 1/β*_lme_*). Heuristically, this allows us to give less weight in the penalty to variables whose standardized regression coefficients are large, because they are more likely to be predictors. When using the adaptive LASSO, we assign a non-negative penalty parameter, λ, to determine how strongly we penalize, or restrict, the magnitude of the PM_2.5_ components regression coefficients. When λ is equal to 0, there is no shrinkage, and the model is just the ordinary mixed-effects regression of the fixed covariates and all components; when it is large enough, there is maximum shrinkage, yielding a model that includes fixed covariates only (all component coefficients equal to 0); when λ takes some value in between, some coefficients are 0, and the model is a penalized model. Components with non-zero coefficients are “selected” by the adaptive LASSO. In this way, the method chooses PM_2.5_ components that may be associated with the outcomes. We ran the models across that range of λs—from no shrinkage to maximum shrinkage—and chose the λ having the smallest Bayesian Information Criterion (BIC) (Schwarz 1978). Last, we used the mixed-effects model with fixed covariates and selected components only, to obtain the estimated effects and corresponding 95% confidence intervals (CIs).

In a sensitivity analysis, we omitted study visits with PM_2.5_ below the 75th percentile of the distribution (12 μg/m^3^).

Data cleaning was performed with SAS 9.3 (SAS Institute Inc.), and data analysis was performed with R 3.1.2 (R Core Team 2015).

## Results

Table 1 summarizes the characteristics of study population. Subjects in this study were elderly men, with a mean (± SD) age of 73 ± 7 years at the first visit. Average SBP and DBP at the first visit were 132 ± 17 mmHg and 76 ± 10 mmHg, respectively.

**Table 1:** Characteristics of subjects in the study.

Variable	First visit (*n* = 718)	All visits (*n* = 1,567)
Mean ± SD
SBP (mmHg)	131.6 ± 16.7	128.1 ± 17.6
DBP (mmHg)	75.9 ± 9.9	71.9 ± 10.3
Age (years)	72.8 ± 6.8	74.7 ± 6.8
BMI (kg/m^2^)	28.2 ± 4.0	28.0 ± 4.1
Education (years)	14.6 ± 2.8	14.6 ± 2.8
*n* (%)
Use of ACE inhibitors	197 (27)	540 (34)
Use of non-ophthalmic beta blockers	213 (30)	554 (35)
Use of calcium channel blockers	104 (14)	265 (17)
Use of diuretics	150 (21)	381 (24)
Use of angiotensin receptor antagonists	36 (5)	124 (8)
Current smokers	28 (4)	47 (3)
Former smokers	488 (68)	1,049 (67)
Two or more drinks per day	143 (20)	299 (19)

PM_2.5_ and component concentrations are shown in Table 2. 7-day moving-average PM_2.5_ across all study visits had a mean of 10 ± 3.7 μg/m^3^, with an interquartile range (IQR) of 4.3 μg/m^3^. S accounted for the largest proportion of the total PM_2.5_ concentration (10.4%), followed by Na (1.9%). The average concentration of Ni was 3.1 ± 2.5 ng/m^3^, and it only accounted for 0.03% of the mass concentration.

**Table 2:** Mean PM_2.5_ mass and component concentrations across all study visits.

Pollutant	Mean ± SD	IQR	Proportion of PM_2.5_ (%)
PM_2.5_ (μg/m^3^)	10.0 ± 3.7	4.3
Component (ng/m^3^)
Fe	68.1 ± 24.2	21.5	0.7
K	39.2 ± 24.6	16.9	0.4
S	1039.1 ± 513.2	554.1	10.4
Al	51.8 ± 27.8	21.1	0.5
Si	76.7 ± 51.1	38.4	0.8
Ni	3.1 ± 2.5	2.5	0.03
V	3.5 ± 2.3	2.6	0.04
Cu	3.5 ± 1.2	1.5	0.04
Zn	11.4 ± 6.0	5.8	0.1
Se	0.2 ± 0.3	0.3	0.002
Na	190.7 ± 72.4	92.8	1.9

**Figure 1:**
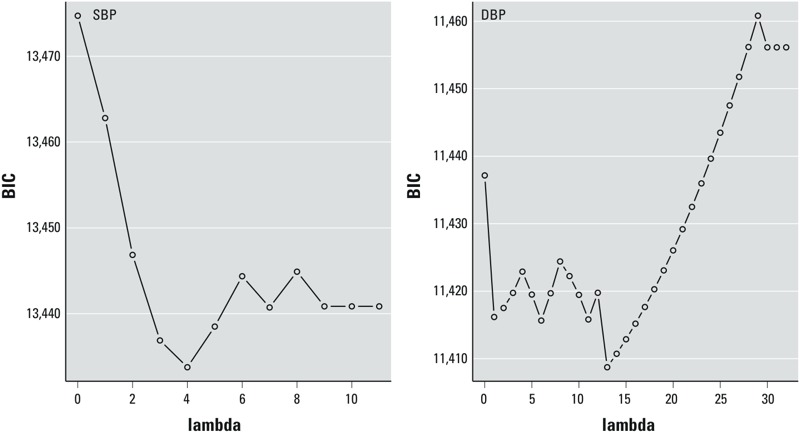
The relationship between BIC, a criterion for model selection and λ (lambda), the adaptive LASSO penalty parameter, for SBP and DBP.

Figure 1 shows the relationship between BIC, a criterion for model selection and λ, the adaptive LASSO penalty parameter. For SBP models, the model with the smallest BIC had λ = 4 and Ni and Na as the only two among the 11 PM_2.5_ components (i.e., K, S, Se, Al, Si, Fe, Ni, V, Cu, Zn, Na) with non-zero coefficients, whereas all component coefficients were zero when λ = 9. For DBP models, the model with the smallest BIC had λ = 13 and Ni as the only component with a non-zero coefficient, whereas all component coefficients were zero when λ = 30.

In models fitted using only the selected components, we found that an IQR increase (2.5 ng/m^3^) in 7-day moving-average Ni was associated with a 2.48-mmHg (95% CI: 1.45, 3.50 mmHg) increase in SBP and a 2.22-mmHg (95% CI: 1.69, 2.75 mmHg) increase in DBP, respectively. To compare with other studies, we also estimated the effects of PM_2.5_ mass: Every 10-μg/m^3^ increase in 7-day moving-average PM_2.5_ was associated with a 1.36-mmHg (95% CI: –1.67, 4.39 mmHg) increase in SBP and a 0.61-mmHg (95% CI: –0.85, 2.07 mmHg) increase in DBP, respectively.

**Figure 2:**
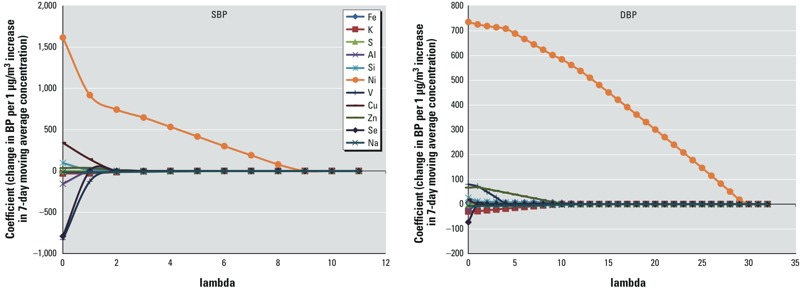
LASSO coefficient paths: plot of coefficient profiles for PM_2.5_ components as a function of λ (lambda).

LASSO coefficient paths for SBP and DBP are shown in Figure 2. Each component coefficient is expressed as the change in mean SBP or DBP per 1-μg/m^3^ increase in the 7-day moving-average concentration of the PM_2.5_ component. Each curve indicates the rate at which the component coefficient shrinks toward zero as λ increases. When λ = 0, all components have non-zero coefficients.

Table 3 shows the comparison of results from the main analysis and the sensitivity analysis that was restricted to data from study visits with PM_2.5_ concentrations below the 75th percentile of the distribution (12 μg/m^3^). We found that the estimated coefficients of Ni for both SBP and DBP in the sensitivity analysis were comparable with those in the main analysis, and their statistical significance remained. That is, Ni was associated with SBP and DBP even when overall PM_2.5_ concentrations were restricted to < 12 μg/m^3^.

**Table 3:** Comparison of estimated coefficients of Ni in the main analysis and in the sensitivity analysis where study visits with 7-day moving-average PM_2.5_ ≥ 12 μg/m^3^ were excluded.

Analysis (no. of visits)	SBP		DBP	
	Coefficient	*p*-Value	Coefficient	*p*-Value
Main analysis (*n* = 1,567)	0.989	< 0.001	0.888	< 0.001
Sensitivity analysis (*n* = 1,201)	1.149	< 0.001	1.104	< 0.001

## Discussion

In this study, we used the adaptive LASSO shrinkage method to choose PM_2.5_ components that might be related to blood pressure in a cohort of elderly men. We found that 7-day moving-average concentrations of Ni and Na were associated with SBP, and 7-day moving-average Ni concentration was also associated with DBP. This association persisted when restricted to data from study visits with PM_2.5_ concentrations < 12 μg/m^3^.

Ni in ambient air is considered a marker of oil combustion; other sources of Ni include coal combustion, nickel metal refining, sewage sludge incineration, and manufacturing facilities [U.S. Environmental Protection Agency (EPA) 2000]. A number of toxicological studies examined the effects of ambient Ni on cardiovascular health. In a mouse model of atherosclerosis, mice had acute changes in heart rate and heart rate variability when exposed to concentrated fine PM (average concentration of Ni was 43 ng/m^3^, and there were Ni peaks at ~ 175 ng/m^3^) (Lippmann et al. 2006). Another animal study showed that Ni inhalation caused a decrease of 75 bpm in maximal heart rate at the concentration of 1.3 mg/m^3^ and a decrease of 100 bpm at 2.1 mg/m^3^ in rats (Campen et al. 2001). Moreover, Ni was reported to induce increases in pulmonary protein leakage and perivascular and peribronchiolar inflammation in both normotensive and spontaneously hypertensive rats that were intratracheal instilled with 1.5 μmol/kg of NiSO_4_•6H_2_O in saline (Kodavanti et al. 2001). A similar study found alterations in heart rate variability (HRV) related to PM exposure were Ni-dependent in spontaneously hypertensive rats after adjustment for HRV responses in control rats (Chuang et al. 2013).

Several epidemiological studies have provided evidence of cardiovascular effects of Ni. A national study conducted in 106 U.S. counties reported that associations between PM_2.5_ concentrations and cardiovascular and respiratory hospitalizations were stronger when Ni was high (Bell et al. 2009). Zanobetti et al. (2009) examined associations of PM_2.5_ with emergency hospital admissions in 26 U.S. communities and found that Ni significantly modified the association between PM_2.5_ mass and hospital admissions for cardiac diseases and myocardial infarctions. A recent study found a significant association between ischemic heart disease mortality and Ni based on data from the American Cancer Society (Lippmann et al. 2013). On the other hand, Zhou et al. (2011) failed to find cumulative effects from lag 0 to lag 2 of Ni in Detroit, Michigan, or Seattle, Washington. A more recent nationwide study that included 75 U.S. cities did not observe any effect modification of Ni in the PM_2.5_–mortality association (Dai et al. 2014).

There are several possible reasons for the differences in these epidemiological studies. First, Ni concentrations are usually lower than the method detection limits, which makes it difficult to determine whether associations are present (Burnett et al. 2000). New York counties had particularly high levels of Ni (a mean of 19.0 ng/m^3^ Ni in New York fine PM vs. a mean of 1.9 ng/m^3^ Ni in national fine PM) due to combustion of residual oil-fired power plants and ocean-going ships (Lippmann et al. 2006). In a reanalysis of the National Morbidity, Mortality, and Air Pollution Study (NMMAPS) data, Dominici et al. (2007) found evidence of effect modification by Ni, which was consistent with the results of Lippmann et al. (2006); however, the effect modification of Ni on the PM–mortality association was much weaker and no longer statistically significant when New York counties were excluded from the analysis. In the two studies that did not find significant associations, Ni had a relatively low level. For example, the national mean concentration of Ni was 2.5 ng/m^3^ in the study by Dai et al. (2014). Given the substantial differences in Ni concentrations, it is conceivable that studies conducted in other places or nationally may not be able to observe the same health effects of Ni as the New York studies did. In our study, Ni had an average concentration of 3.1 ng/m^3^, which was higher than the national mean but still much lower than New York levels (Dominici et al. 2007; Lippmann et al. 2006). Furthermore, it is possible that Ni interacts with other PM components to pose an increased risk to health. Campen et al. (2001) reported evidence of a synergistic interaction between Ni and V, both of which are markers of PM from oil combustion. Hence, the heterogeneous composition of PM in different locations might lead to different estimated effects of Ni.

Na also was selected, in addition to Ni, when the adaptive LASSO method was applied to identify PM_2.5_ components associated with SBP. There is limited literature on the effects of ambient Na on cardiovascular health. Zanobetti et al. (2009) documented that Na^+^ modified the relationship between PM_2.5_ and emergency hospital admissions for cardiac diseases.

In the study, the maximum level of 7-day moving-average PM_2.5_ concentration was 34.3 μg/m^3^, whereas daily PM_2.5_ peaked at 44.8 μg/m^3^ with a 99th percentile of 34 μg/m^3^. Hence, we identified associations in a study population that was usually exposed to PM_2.5_ concentrations below the current U.S. EPA daily ambient standard of 35 μg/m^3^ (U.S. EPA 2012). Associations with Ni were similar when we excluded observations with 7-day moving-average PM concentration ≥ 12 μg/m^3^. Our findings may suggest stricter air quality standards.

To date, many studies have investigated the biological mechanisms of the adverse effects of inhalation exposures to PM on cardiovascular diseases. Brook et al. (2010) summarized three potential pathways: *a*) inducing pulmonary oxidative stress and inflammation via the release of proinflammatory mediators or vasculoactive molecules; *b*) interacting with lung receptors or nerves to perturb systemic autonomic nervous system balance or heart rhythm; or *c*) PM or PM components being transmitted into the systemic circulation. Metals are typical PM components. It has been documented that metals can enhance lung inflammation and injury (Ghio and Devlin 2001; Schaumann et al. 2004), which may be attributed to the metal-catalyzed oxygen stress via non-nitric oxide pathways (Dye et al. 1997). Nevertheless, mechanisms of cardiovascular effects of Ni have not been fully established. Previous studies have shown that metals in particles (e.g., Ni, V) could induce the activation of transcription factor NF-κB (nuclear factor κB; a family of proteins that regulates DNA transcription in cellular responses such as immune, inflammatory response, and apoptosis), cell apoptosis, and cell cycle regulation (Chen and Shi 2002; Goebeler et al. 1995; Quay et al. 1998). Although the clinical relevance is unclear, our finding that an IQR increase in Ni was associated with a 2.48/2.22-mmHg increase in blood pressure may imply elevated risks of cardiovascular outcomes induced by Ni.

The major strengths in the study are as follows: First, we used a novel approach, the adaptive LASSO, to investigate the relationship between PM_2.5_ components and health outcomes. This method has advantages over conventional approaches. Typically, researchers examined effects of components by including all components in models or by using conventional selection procedures, such as stepwise selection. Linear regression with all components included may fail to detect any association because the collinearity among components reduces power, and conventional selection methods make no guarantee to select the right variables asymptotically. Second, to our knowledge, this is the first longitudinal cohort study to examine the effects of PM-related metals on blood pressure. The study population was geographically stable, well described, and followed up since enrollment in 1963. Third, we had daily concentrations of PM metals for > 10 years. In previous studies, especially large national/multi-city studies, researchers usually used data from the U.S. EPA Air Quality System that was sampling PM components every third or sixth day (Dai et al. 2014; Krall et al. 2013; Zanobetti et al. 2009) and hence had to face the challenge in lack of data.

On the other hand, there are several limitations in the study. Due to the use of stationary measures of PM_2.5_ components, we were unable to capture the personal exposures of our subjects. Another limitation of our study is the potential measurement errors in blood pressure, because blood pressure was measured only once at each study visit. Last, because the study population was limited to elderly men, most of whom were Caucasian, our findings cannot be directly generalized to women, younger men, or more diverse populations of elderly men. Subjects voluntarily continue to participate in the ongoing NAS study, so there may be volunteer bias if healthier people are more likely to participate. Also, there would be survivor bias if people who stay in the study are healthier than other people.
